# Machine-Learning-Based Prediction of Preterm Birth in Women with Huge Uterine Fibroids: A Stratified Cohort Analysis

**DOI:** 10.3390/diagnostics16142242

**Published:** 2026-07-17

**Authors:** Simon Shenhav, Eyal Sadeh, Yaniv S. Ovadia, Amit Shenhav, Eyal Y. Anteby, Abigail Paradise Vit

**Affiliations:** 1 Obstetrics and Gynecology Division, Barzilai University Medical Center, Ashkelon 7830604, Israel; saimons@bmc.gov.il (S.S.); eyala@bmc.gov.il (E.Y.A.); 2 Faculty of Health Sciences, Ben-Gurion University of the Negev, Beersheba 8410501, Israel; 3 Department of Information Systems, The Max Stern Emek Yezreel College, Emek Yezreel 1930600, Israel; eyals@yvc.ac.il (E.S.); abigailp@yvc.ac.il (A.P.V.); 4 BIG DATA Research Unit, Barzilai University Medical Center, Ashkelon 7830604, Israel; 5 Ruth and Bruce Rappaport Faculty of Medicine, Technion—Israel Institute of Technology, Haifa 3200003, Israel; amit.sh@campus.technion.ac.il; 6 Rambam Health Care Campus, Haifa 3109601, Israel

**Keywords:** preterm birth, uterine fibroids, machine learning, risk prediction

## Abstract

**Background/Objectives**: Preterm birth remains a major cause of neonatal morbidity and mortality, and risk stratification in pregnancies with uterine fibroids is limited. This study evaluated whether detailed phenotyping of huge uterine fibroids provides predictive information. **Methods**: This retrospective single-center study included 192 singleton pregnancies: 64 with a huge uterine fibroid (maximum diameter ≥ 10 cm) and 128 fibroid-free controls. We analyzed the full and fibroid-only cohorts. Machine learning (ML) models were compared across full fibroid-feature, alternative fibroid-feature, non-fibroid, and clinical benchmark configurations. **Results**: In validation analyses, the alternative fibroid-feature configuration was selected as the best-performing configuration in both cohorts. The best validation models were Random Forest for the full cohort and Logistic Regression for the fibroid-only cohort, achieving F1-scores of 0.67 and 0.80 and areas under the receiver operating characteristic curve (AUCs) of 0.94 and 0.90, respectively. On the held-out test set, models achieved F1-scores of 0.50 and 0.57, with AUCs of 0.82 and 1.00, respectively; uncertainty and calibration remained limited by the very small number of positive events. SHapley Additive exPlanations analysis showed that fibroid-related variables contributed to model output. **Conclusions**: Detailed fibroid phenotyping may add predictive information beyond clinical variables, but these exploratory findings require further validation in larger cohorts.

## 1. Introduction

Preterm birth, defined as delivery before 37 completed weeks of gestation, remains a major cause of neonatal morbidity and mortality worldwide [1]. Global estimates suggest that roughly 1 in 10 births occur preterm, underscoring the substantial population burden and the need for better risk stratification [1]. Because the causal pathways are heterogeneous and include both spontaneous and medically indicated preterm birth, prediction is clinically challenging [1,2]. Early identification of women at risk is important because it can support surveillance, counseling, and timely preventive or referral strategies. Uterine fibroids are common benign smooth-muscle tumors of the female reproductive tract and are often detected incidentally on obstetric ultrasound [3,4]. Although often asymptomatic during pregnancy, fibroids have been associated with preterm birth, placental complications, higher rates of cesarean delivery, and postpartum hemorrhage [3,4]. Proposed mechanisms include distortion of the uterine cavity, altered myometrial vascularity, impaired uterine contractility, and interference with placentation [5,6]. Importantly, accumulating evidence suggests that fibroid burden, size, type, and anatomical location may modify these risks [3,7]. However, evidence regarding huge fibroids remains limited. Although no uniform definition exists, the terms large, very large, and ‘huge’ have been used inconsistently to describe fibroids exceeding 10 cm. In the present study, ‘huge’ refers to fibroids measuring ≥10 cm, a threshold supported by evidence demonstrating a size-dependent increase in adverse obstetric outcomes, with fibroids >10 cm showing the highest rates of several adverse pregnancy outcomes [8].

Despite this, most preterm birth prediction models either do not include fibroids or encode them only as a binary diagnosis, without accounting for detailed fibroid phenotyping [9,10,11]. A growing body of literature has applied machine learning (ML) techniques to preterm birth prediction in general obstetric cohorts [10,12,13,14]. However, only a small subset of this literature includes uterine fibroids/myomas as predictors [4,11,13], typically encoding them as a binary diagnosis or complication variable. Consequently, fibroid-related heterogeneity is not well represented in existing preterm birth prediction models, and the predictive role of detailed fibroid characteristics remains poorly understood.

To address this gap, we evaluated ML models for preterm birth prediction using detailed fibroid phenotyping in two complementary cohorts: a full cohort including pregnancies with and without fibroids, and a fibroid-only cohort restricted to women with huge uterine fibroids. Within each cohort, four feature-set configurations were assessed: a full fibroid-feature ML configuration, an alternative fibroid-feature ML configuration, a non-fibroid configuration excluding all direct and derived fibroid-related predictors, and a conventional clinical benchmark. This design allowed us to examine whether fibroid-related characteristics provide incremental predictive information beyond non-fibroid predictors and simpler clinical models. We further applied SHapley Additive exPlanations (SHAP) [14] to assess the contribution of fibroid-related, maternal, obstetric, demographic, and pregnancy-related factors to model performance and interpretability.

## 2. Materials and Methods

We conducted a retrospective cohort study. The dataset comprised 192 individual records, each representing a unique pregnancy, and included variables encompassing demographic, clinical, and fibroid-related information. Data were collected at the Obstetrics and Gynecology Division, Barzilai University Medical Center, Ashkelon (BUMCA), Israel, covering the period from January 2010 to December 2023.

### 2.1. Participants, Setting, and Design

The study group (*n* = 64) included all women with singleton pregnancies who had a single huge uterine fibroid with a measured maximum fibroid diameter of ≥10 cm identified during routine obstetric ultrasonography in the first or second trimester, and who subsequently delivered at BUMCA. All fibroid-related variables and candidate predictors were derived from data available during the first or second trimester, prior to delivery. Women without documented uterine fibroids who delivered at BUMCA during the same study period were randomly selected as controls in a 1:2 ratio (*n* = 128) relative to the index cases.

To minimize confounding factors potentially affecting timing of delivery, thus isolating the effect of a single fibroid, exclusion criteria for both groups included previous preterm birth, multiple gestation, maternal infections, hypertensive disorders, and prior cervical surgery [1,2,3,4,7], as well as uterine anomalies, the presence of multiple fibroids, chronic hypertension, autoimmune disease, and malignancy.

### 2.2. Study Protocol

Demographic and clinical characteristics, including maternal age, body mass index (BMI), gravidity, parity, education level, smoking status, and past medical history, were collected from the electronic medical records of the participants.

Transvaginal or abdominal ultrasound examinations were performed by experienced fellowship-trained ultrasonographers using high-resolution machines: Voluson 730 PRO and Voluson E8 Expert (GE Healthcare Zipf, Tiefenbach, Austria). Ultrasonographic data included detailed assessment of the fibroid in three key dimensions: maximum diameter, myometrial relationship, and anatomical location. Fibroids were classified according to the International Federation of Gynecology and Obstetrics (FIGO) leiomyoma classification system based on the earliest routine first or second trimester ultrasound examination. Lesions were categorized as intramural (FIGO types 3–5, with ≥50% of the lesion located within the myometrium) or subserosal (FIGO types 6–7, with <50% myometrial involvement). No submucosal fibroids were observed in this cohort.

Anatomical location was recorded separately to allow subgroup analysis comparing high-risk locations, including the lower uterine segment (LUS), cervical, and previa locations, with lower-risk locations. All ultrasound images were reviewed by a second independent expert sonographer to confirm classification.

Image review and classification were performed under the supervision of a senior obstetrician-gynecologist with formal ISUOG fellowship training in obstetric and gynecological ultrasound.

The primary outcome assessed in this study was preterm birth, defined as gestational age at delivery before 37 completed weeks of gestation. Additional obstetric and neonatal characteristics were collected from the medical records for descriptive purposes.

**Figure 1:**
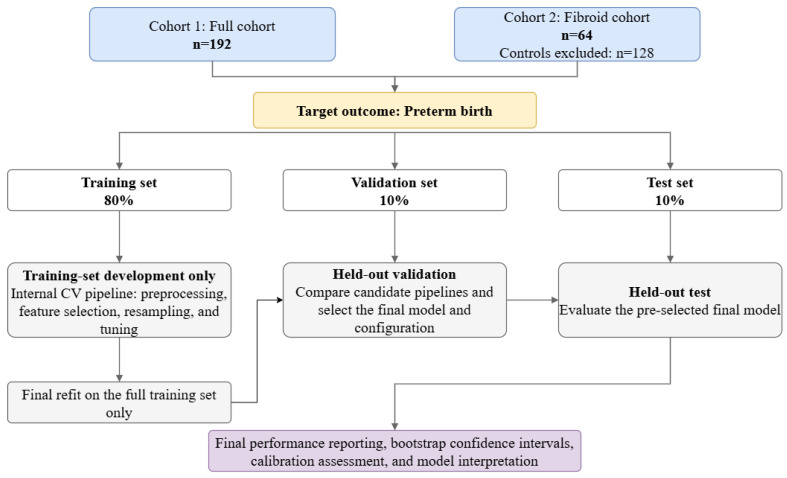
Study workflow and cohort derivation for ML prediction of preterm birth. The target outcome, preterm birth, was modeled in two cohorts: Cohort 1, including the full study population, and Cohort 2, restricted to pregnancies with huge uterine fibroids after exclusion of controls. For each cohort, data were split into training, validation, and test subsets using an 80%/10%/10% split. Model development was performed only within the training set, including internal cross-validation, preprocessing, feature selection, resampling, and hyperparameter tuning. Candidate pipelines were compared on the held-out validation set, and only the pre-selected final model was evaluated on the held-out test set. Final reporting included performance metrics, bootstrap confidence intervals, calibration assessment, and SHAP-based interpretability.

Figure 1 summarizes the end-to-end study design, from cohort construction through model development and evaluation for preterm birth. The figure highlights which cohorts were used for the prediction task and outlines the main steps of the ML pipeline. Each component of this workflow is described in detail in the following subsections. The ML workflow and reporting were aligned with TRIPOD+AI recommendations [15] for clinical prediction models using ML methods. All analyses and ML procedures were performed using Python version 3.14. The main packages and versions used for preprocessing, modeling, resampling, feature selection, calibration, and interpretability are provided in Supplementary Table S1.

### 2.3. Cohort Definitions, Features and Target Outcome

#### 2.3.1. Cohort Definitions

The study dataset comprised two primary groups: a control group without uterine fibroids (*n* = 128) and a fibroid group with huge uterine fibroids (*n* = 64), together forming the full cohort (Cohort 1; *n* = 192).

Based on these data, two analysis cohorts were defined (Figure 1):

- Cohort 1 (full cohort; *n* = 192) included all eligible pregnancies from both groups.
- Cohort 2 (fibroid-only cohort; *n* = 64) included only pregnancies with huge uterine fibroids (i.e., after excluding controls without fibroids; *n* = 128).

#### 2.3.2. Target Outcome

**Figure 2:**
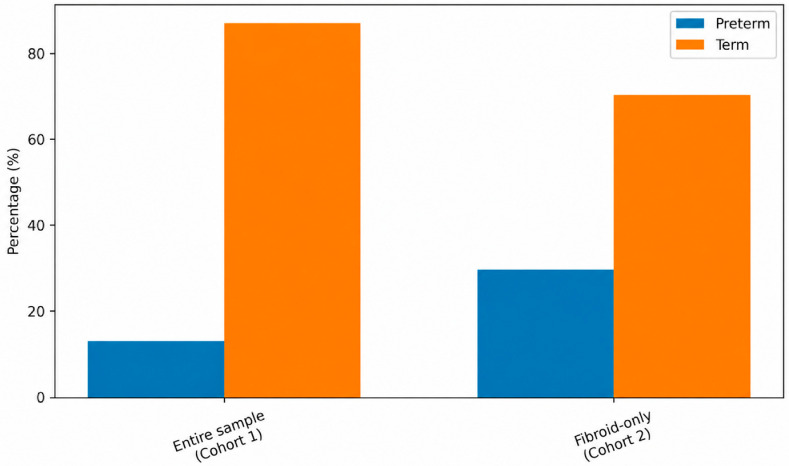
Distribution of preterm birth across the study cohorts. The percentage of preterm and term births is shown for the entire sample and the fibroid-only cohort.

The preterm birth outcome was modeled using Cohort 1 (full cohort) and Cohort 2 (fibroid-only cohort) to evaluate prediction performance both in the combined population (controls + fibroid pregnancies) and within the fibroid group specifically. This design enabled outcome-specific modeling both in the broader cohort and in the fibroid-only subgroup. Figure 2 illustrates the distribution of the binary outcome across the study cohorts. Pregnancies complicated by huge fibroids showed higher rates of preterm birth than the corresponding full-sample cohort.

### 2.4. Candidate Features

Feature names were standardized, and categorical values were harmonized to minimize fragmented or inconsistent labeling. To avoid information leakage, only predictors available prior to the outcome event were included, whereas post-delivery and outcome-derived variables were excluded from the analysis.

Regarding missing data, no variables contained missing values except for fibroid-related fields. These missing entries were structurally expected in pregnancies without fibroids and were therefore interpreted as structural missingness due to the absence of fibroids. Accordingly, indicator variables were created to distinguish between true absence of fibroids and observed fibroid measurements, and missing values in fibroid-specific fields were handled consistently on this basis.

All variables were then converted to clinically appropriate data types, with count variables stored as integers and continuous measures as floating-point values. In addition, a set of interpretable derived features was generated to support the predictive modeling framework.

Table 1 summarizes the candidate predictors considered in the study, grouped into five clinically relevant domains: fibroid-related characteristics, maternal anthropometrics, obstetric history, maternal demographics, and current pregnancy uteroplacental insufficiency risk factors.

Fibroid-related variables included study group (control vs fibroid-only cohort), fibroid presence (yes/no), maximal fibroid diameter, fibroid type, and anatomical location. Maximum fibroid diameter was recorded as a continuous variable in centimeters. In addition, several categorical representations of fibroid size were examined. First, maximum fibroid diameter was divided into tertile-based categories: no fibroid, 10.0–10.1 cm, 10.1–12.1 cm, and >12.1 cm. Second, a cutoff-based variable was created using the upper tertile threshold, classifying participants as having no fibroid, a fibroid below or equal to 12.1 cm, or a fibroid larger than 12.1 cm. Third, a median-based variable was created by classifying participants as having no fibroid, a fibroid below or equal to the median size, or a fibroid above the median size.

Additional fibroid descriptors included fibroid type (no fibroid, subserosal, intramural, cervical, or previa) and anatomical location (no fibroid, fundus, lower uterine segment, previa, posterior wall, or anterior wall). Fibroid type and location were incorporated as distinct variables because different fibroid subtypes may influence pregnancy outcomes through distinct pathways. Subserosal fibroids were defined as fibroids located along the outer uterine surface, intramural fibroids as fibroids embedded within the myometrium, and cervical fibroids as fibroids originating from the cervix. Fibroid previa referred to fibroids located within the lower uterine segment adjacent to or overlying the internal cervical os. Anatomical location was classified according to the uterine region in which the fibroid was identified to assess whether fibroid location independently influences preterm birth risk. Collectively, these fibroid characteristics were included because fibroid size, type, and location may differentially affect uterine distortion, placentation, myometrial architecture (and thus, uterine contractility), and mechanical obstruction during pregnancy, each of which may contribute independently to preterm birth risk.

Maternal anthropometric variables included height, weight, and BMI. BMI was analyzed both as a continuous variable and as a categorical variable. BMI categories were defined according to World Health Organization criteria [16] as underweight (<18.5 kg/m^2^), normal weight (18.5–24.9 kg/m^2^), overweight (25.0–29.9 kg/m^2^), and obese (≥30.0 kg/m^2^). In addition, obesity status was defined dichotomously as obese (BMI ≥ 30.0 kg/m^2^) versus non-obese (BMI < 30.0 kg/m^2^).

Obstetric variables included gravidity, parity, and previous abortions as count variables, along with parity category [17] (nulliparous if parity = 0, primiparous if parity = 1, and multiparous if parity ≥ 2) and high gravidity as binary indicators (1 if gravidity > 3, otherwise 0).

Maternal demographic variables included maternal age as both a continuous and grouped variable, categorized as adolescent (<20 years), young adult (20–34 years), advanced maternal age (35–39 years), and very advanced maternal age (≥40 years), as well as education level (secondary vs. tertiary). Current pregnancy risk factors included smoking status (current smoker vs. non-smoker) and the presence of gestational diabetes mellitus (GDM).

Categorical predictors were transformed into binary indicator (dummy) variables using one-hot encoding to enable their inclusion in the ML models.

### 2.5. Redundancy Assessment and Feature-Set Configurations

Before model development, we assessed redundancy among candidate predictors using pairwise dependency measures selected according to variable type [18]. Spearman rank correlation was used for continuous–continuous pairs, Cramer’s V for categorical–categorical pairs, and the phi coefficient for binary–binary pairs. The redundancy results were reviewed together with clinical considerations, and when variables represented overlapping, derived, or alternative encodings of the same underlying construct, we retained the most clinically interpretable or analytically appropriate representation and removed alternative encodings before model training.

To evaluate the contribution of fibroid-related predictors and the robustness of the modeling strategy, four feature-set configurations were defined before model development:

1. Full fibroid-feature ML: The main ML analysis, including retained clinical, demographic, obstetric, and fibroid-related predictors after excluding redundant or derived variables.
2. Alternative fibroid-feature ML: A sensitivity analysis using the same non-fibroid predictors as the full configuration, but restricting the fibroid-related component to a smaller clinically non-redundant set of fibroid variables.
3. Non-fibroid ML: An ablation analysis in which all direct and derived fibroid-related predictors were removed, to assess the added predictive value of fibroid-related information.
4. Clinical benchmark: A conventional statistical comparison based on a limited pre-specified clinical feature set.

The full fibroid-feature, alternative fibroid-feature, and non-fibroid configurations were analyzed using the ML pipeline described below. The clinical benchmark configuration was analyzed separately and did not include feature selection, resampling, or hyperparameter tuning.

The resulting retained and excluded feature sets for each analysis configuration are described in Section 3.2.

For the clinical benchmark configuration, we evaluated two pre-specified statistical models in each cohort: multivariable logistic regression without regularization [19] and Firth’s penalized logistic regression [20]. These models were developed separately from the ML pipeline and did not use feature selection, cross-validation-based tuning, oversampling, or ML-based hyperparameter optimization. The clinical benchmark feature set was defined a priori based on established clinical relevance [21] and included previous abortions, smoking status, parity, gestational diabetes mellitus, and BMI. The same train/validation/test split used for the ML analyses was applied to the clinical benchmark models, and performance was evaluated on the held-out validation set using the same metrics as the ML models.

### 2.6. Data Splitting and Events-per-Variable Assessment

For each cohort, the dataset was split into training, validation, and test subsets using a stratified 80%/10%/10% split to preserve the distribution of the target outcome across all subsets. A fixed random seed of 42 was used to ensure reproducibility. The training set was used exclusively for internal model development, including preprocessing, feature selection, resampling, hyperparameter tuning, and final model refitting. The held-out validation set was used only for comparing candidate pipelines and selecting the final model configuration. The held-out test set remained untouched throughout model development and was used only once for final evaluation of the pre-selected model.

To quantify the number of outcome events relative to model complexity, we calculated events-per-variable (EPV) estimates for each cohort and analysis configuration. EPV was calculated as the number of preterm birth events divided by the number of candidate predictors. For each cohort, we reported the total sample size, total number of preterm birth events, and the number of events in the training, validation, and test subsets. We also reported the number of candidate predictors before redundancy reduction, the number of retained predictors after redundancy-based feature reduction, and the number of final selected features after the full-training-set refit.

Training-set EPV was calculated as the number of preterm birth events in the training set divided by the number of retained candidate predictors before feature selection. This definition was used because feature selection was data-driven and therefore could not be used to justify the initial event-to-predictor ratio. These analyses were added because of the small sample size and limited number of preterm birth events, particularly in the fibroid-only cohort.

### 2.7. Model Training, Validation-Based Model Selection, and Final Test-Set Assessment

We implemented a supervised ML pipeline separately for each cohort and feature-set configuration. For the full fibroid-feature, alternative fibroid-feature, and non-fibroid ML configurations, three model families were evaluated: Logistic Regression (LR), Random Forest (RF), and Light Gradient Boosting Machine (LightGBM). LR was used as a linear baseline with sparsity-inducing L1 (LASSO) and shrinkage L2 (ridge) penalties [22]. RF was used as an ensemble of randomized decision trees aggregated by bagging [23]. LightGBM was used as a histogram-based gradient boosting decision-tree method designed for efficient learning from tabular data [24].

Candidate models were trained and tuned using stratified 5-fold cross-validation performed only within the training set. Model development followed a leakage-controlled sequence. Within each cross-validation fold, preprocessing transformations were fitted only on the fold-training data and then applied to the corresponding fold-validation data.

Feature selection was also performed only within the fold-training data. Four complementary feature-selection methods were used: Mutual Information (MI), which quantified how much each feature reduced uncertainty about the target outcome [25]; L1-regularized logistic regression (LASSO), which promoted sparsity by shrinking weak or redundant predictors toward zero [22]; RF feature importance, which quantified each feature’s contribution within an ensemble of decision trees [23]; and Recursive Feature Elimination (RFE), which iteratively removed the least informative features according to an estimator-specific importance criterion [26].

Each feature-selection method was run independently within the fold-training data. A consensus-based rule was then applied within each fold, retaining features selected by at least two of the four methods. The resulting fold-level consensus feature set was applied to both the fold-training and fold-validation data within that fold. Because feature selection was repeated independently in each fold, selected feature subsets were allowed to vary across folds and were documented as part of the feature-selection stability analysis.

Class imbalance was addressed only within the training data, while preserving the original outcome distribution in the fold-validation, held-out validation, and held-out test sets. For each ML configuration, three training strategies were evaluated: the original imbalanced training data, random oversampling (ROS), and SMOTENC [27]. During cross-validation, resampling was applied only to the fold-training data after preprocessing and feature selection. Resampling was never applied to fold-validation data, held-out validation data, or held-out test data.

Hyperparameter tuning was performed within the training set using randomized search with 10 iterations and stratified cross-validation. For the ML models, scikit-learn’s RandomizedSearchCV was used with score-based model ranking. After tuning, each pipeline was refitted on the full training set only, repeating preprocessing, feature selection, and resampling where applicable. For this final refit, resampling was applied only to the full training set and never to the held-out validation or test sets.

Candidate pipelines were compared on the held-out validation set, which was used only for model and configuration selection. After the final model and feature-set configuration were selected for each cohort, only the selected final models were evaluated on the held-out test set. The primary model-selection criterion was F1-score for the preterm birth class, reflecting the clinical importance of identifying preterm birth cases. When F1-scores were tied, the area under the receiver operating characteristic curve (AUC) was used as a secondary criterion. Additional reported metrics included accuracy, precision, recall, Cohen’s kappa, and Brier score.

Because the dataset was small and the number of preterm birth events in the held-out test subsets was limited, additional uncertainty and calibration analyses were performed for the final selected models. Bootstrap confidence intervals [28] were calculated only for the final selected model in each cohort, using the held-out test-set predictions. Models were not retrained during bootstrap. A stratified bootstrap procedure with 2000 iterations was used to preserve the original number of positive and negative cases in each resampled test set. In each bootstrap sample, the AUC, F1-score, recall, precision, accuracy, Cohen’s kappa, and Brier score were calculated when feasible. The 95% confidence intervals were defined using the 2.5th and 97.5th percentiles of the bootstrap distribution. The classification threshold was kept fixed and was not re-optimized using the test set.

Calibration assessment [29] was also performed only for the final selected model in each cohort using the held-out test-set predicted probabilities. Calibration was assessed using Brier score, calibration plots comparing mean predicted probability with the observed fraction of preterm birth cases, and calibration slope and intercept when numerically feasible. Because the test sets were small and included very few positive events, calibration plots were generated using a limited number of quantile-based bins and were interpreted cautiously.

### 2.8. Model Interpretability Using SHAP

We conducted post hoc model interpretability analysis using SHAP to obtain both global and local explanations of the selected models for preterm birth prediction. SHAP is a game-theoretic explanation method that attributes each prediction to individual features by computing their marginal contributions to the model output. SHAP values were calculated to quantify each feature’s contribution to the model output, enabling visualization of overall feature importance across each cohort as well as case-level explanations for individual predictions, thereby supporting clinical interpretability [14].

SHAP analysis was performed using the final selected models for each cohort. For each selected model, the background set was sampled from the original training split, whereas the explanation set used for the SHAP summary and waterfall plots was the original held-out validation split.

## 3. Results

### 3.1. Cohort Characteristics, Outcome Events, and Events-per-Variable Assessment

The descriptive characteristics of the study cohorts are presented in Appendix A, Table A1. Cohort 1 included the full study population of 192 pregnancies, of which 25 were complicated by preterm birth. Cohort 2 included 64 pregnancies with huge uterine fibroids, of which 19 were complicated by preterm birth. The number of preterm birth events was small in both cohorts, particularly after the stratified train/validation/test split.

Supplementary Table S2 presents the number of preterm birth events and non-events in the total cohort and in each data subset. In Cohort 1, the training, validation, and test subsets included 19, 3, and 3 preterm birth events, respectively. In Cohort 2, the corresponding subsets included 15, 2, and 2 preterm birth events. These event counts emphasize the limited number of positive cases available for model development, validation-based model selection, and final test-set evaluation.

Events-per-variable estimates are also reported in Supplementary Table S2. After redundancy-based feature reduction, training-set EPV remained low across all configurations. In Cohort 1, training-set EPV ranged from 1.58 in the full fibroid-feature ML configuration to 3.80 in the clinical benchmark configuration. In Cohort 2, training-set EPV ranged from 1.25 in the full fibroid-feature ML configuration to 3.00 in the clinical benchmark configuration. Overall, the EPV results indicate that model performance should be interpreted cautiously given the limited number of outcome events relative to the number of candidate predictors.

### 3.2. Redundancy Assessment

Pairwise redundancy assessment results are reported in Supplementary Table S3. Several predictors represented overlapping, derived, or alternative encodings of the same underlying clinical information. The strongest redundancy was observed between the study group variable and several fibroid-related categorical variables, including fibroid size tertile, fibroid location category, and fibroid type. Additional high redundancy was observed among alternative parity, maternal-age, and anthropometric encodings.

Based on these findings, redundant or derived variables were removed before model development. Maximum fibroid diameter was retained as the primary continuous measure of fibroid size, whereas tertile-based, cutoff-based, and median-based fibroid-size variables were excluded from the revised modeling configurations. Similarly, BMI was retained as the primary anthropometric measure, while maternal weight, BMI category, and obesity status were excluded to avoid duplicate BMI-related information. Gravidity, high gravidity, parity category, and maternal age group were also excluded in favor of more direct or clinically interpretable representations.

The resulting feature inclusion and exclusion rules for each analysis configuration are summarized in Supplementary Table S4. After redundancy-based feature reduction, the full fibroid-feature ML configuration retained the broadest candidate predictor set, including non-redundant fibroid-related, maternal anthropometric, obstetric, demographic, and current pregnancy risk-factor variables. This configuration included fibroid presence where informative, maximum fibroid diameter, fibroid type, and fibroid anatomical location, together with maternal height, BMI, parity, previous abortions, maternal age, education level, smoking status, and GDM.

The alternative fibroid-feature ML configuration retained the same non-fibroid predictors but restricted the fibroid-related component to a smaller, clinically non-redundant set, consisting of fibroid presence where informative, maximum fibroid diameter, and fibroid location category. The non-fibroid ML configuration excluded all direct and derived fibroid-related predictors and retained only non-fibroid maternal, obstetric, demographic, and pregnancy-related predictors. The clinical benchmark configuration used a limited pre-specified clinical feature set, consisting of BMI, parity, previous abortions, smoking status, and GDM.

Overall, after redundancy assessment and before feature selection, the full fibroid-feature configuration retained 12 candidate predictors in both cohorts; the alternative fibroid-feature ML configuration retained 11 predictors in Cohort 1 and 10 in Cohort 2; the non-fibroid configuration retained eight predictors; and the clinical benchmark retained five predictors.

### 3.3. Feature Selection

Feature selection was performed independently within each cross-validation fold to avoid information leakage from validation or test data. For the clinical benchmark configuration, feature selection was not applied; the five pre-specified clinical predictors were retained by design.

After redundancy-based feature reduction, the full fibroid-feature ML configuration included fibroid presence, maximum fibroid diameter, fibroid type, fibroid anatomical location, and non-fibroid maternal, obstetric, demographic, and pregnancy-related predictors. The alternative fibroid-feature ML configuration retained the same non-fibroid predictors but used maximum fibroid diameter and fibroid location category as the main fibroid descriptors. The non-fibroid ML configuration excluded all direct and derived fibroid-related variables and retained only maternal height, BMI, parity, previous abortions, maternal age, education level, smoking status, and GDM. The clinical benchmark configuration was restricted to BMI, parity, previous abortions, smoking status, and GDM.

After the full-training-set refit, the number of final selected features differed across cohorts and feature-set configurations. In Cohort 1, 10 of 12 retained candidate predictors were selected in the full fibroid-feature ML configuration; smoking status and GDM were not retained. In the alternative fibroid-feature ML configuration, all 11 candidate predictors were retained. In the non-fibroid ML configuration, all eight candidate predictors were retained.

In Cohort 2, seven of 12 retained candidate predictors were selected in the full fibroid-feature ML configuration; fibroid presence, BMI, previous abortions, education level, and smoking status were not retained. In the alternative fibroid-feature ML configuration, nine of 10 candidate predictors were retained, with education level not retained. In the non-fibroid ML configuration, all eight candidate predictors were retained. The clinical benchmark retained all five pre-specified predictors in both cohorts.

Overall, fibroid-related variables remained part of the final selected feature sets in the fibroid-feature configurations, supporting their relevance to the prediction task. The exact final selected predictors for each cohort and configuration are provided in Supplementary Table S5. Feature-selection results and fold-level performance metrics across cross-validation folds are reported in Supplementary Table S6.

### 3.4. Validation Performance Across Feature-Set Configurations

**Figure 3:**
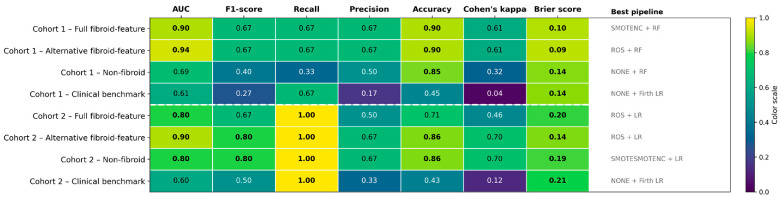
Validation performance of the best-performing pipeline for each cohort and feature-set strategy. The heatmap presents the best-performing validation pipeline for each cohort and feature-set strategy. Rows correspond to the full fibroid-feature model, the alternative fibroid-feature ML configuration, the non-fibroid model, and the clinical benchmark model. Columns show validation performance metrics, including AUC, F1-score, recall, precision, accuracy, Cohen’s kappa, and Brier score. The “Best pipeline” column reports the selected model and resampling strategy for each configuration. For all metrics except the Brier score, higher values indicate better performance. For the Brier score, lower values indicate better probabilistic accuracy/calibration; therefore, the color scale for the Brier score is reversed, while the displayed values represent the original Brier scores.

The best hyperparameters selected for each ML pipeline are presented in Supplementary Table S7. Held-out validation performance for all candidate pipelines is provided in Supplementary Table S8. Figure 3 summarizes the best-performing validation pipeline for each cohort and feature-set strategy.

In Cohort 1, the alternative fibroid-feature ML configuration was selected as the best-performing validation configuration according to the predefined model-selection criterion. The best-performing pipeline was RF with ROS, with an AUC of 0.94, F1-score of 0.67, recall of 0.67, precision of 0.67, accuracy of 0.90, Cohen’s kappa of 0.61, and Brier score of 0.09. The full fibroid-feature ML configuration achieved the same F1-score, recall, precision, accuracy, and Cohen’s kappa, but had a lower AUC of 0.90 and a higher Brier score of 0.10. Therefore, the alternative fibroid-feature configuration was selected based on the AUC tie-breaking rule. Both fibroid-feature configurations outperformed the non-fibroid ML and clinical benchmark configurations in overall validation profile.

In Cohort 2, the alternative fibroid-feature ML configuration was also selected as the best-performing validation configuration. The best-performing pipeline was LR with ROS, with an AUC of 0.90, F1-score of 0.80, recall of 1.00, precision of 0.67, accuracy of 0.86, Cohen’s kappa of 0.70, and Brier score of 0.14. This configuration outperformed the full fibroid-feature ML configuration, which achieved an AUC of 0.80 and F1-score of 0.67. Although the non-fibroid ML configuration achieved the same F1-score, recall, precision, accuracy, and Cohen’s kappa as the alternative fibroid-feature configuration, it had a lower AUC of 0.80 and a higher Brier score of 0.19. Therefore, the alternative fibroid-feature configuration was selected based on the AUC tie-breaking rule.

Overall, validation results suggested that fibroid-related predictors contributed additional predictive information, particularly when comparing the fibroid-feature configurations with the non-fibroid and clinical benchmark configurations.

### 3.5. Final Selected Models: Test Performance, Uncertainty, and Calibration

#### 3.5.1. Final Held-Out Test Performance

The test set was evaluated only after the final model and feature-set configuration had been selected using the held-out validation set.

**Figure 4:**
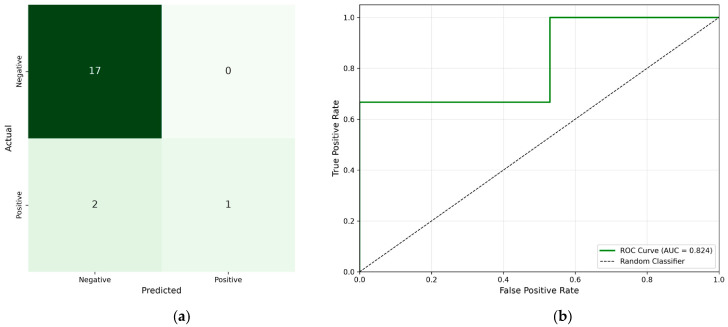
Performance of the final alternative fibroid-feature RF model for Cohort 1, as evaluated on the held-out test set. (**a**) Confusion matrix showing 17 true negatives, 1 true positive, 0 false positives, and 2 false negatives. (**b**) ROC curve demonstrating the model’s discriminative performance on the test set, with an AUC of 0.82.

In Cohort 1, the final selected model was the alternative fibroid-feature RF model trained with ROS. On the held-out test set, this model achieved an AUC of 0.82, F1-score for the preterm birth class of 0.50, recall of 0.33, precision of 1.00, accuracy of 0.90, Cohen’s kappa of 0.46, and Brier score of 0.11. The confusion matrix in Figure 4 shows 17 true negatives, one true positive, zero false positives, and two false negatives.

**Figure 5:**
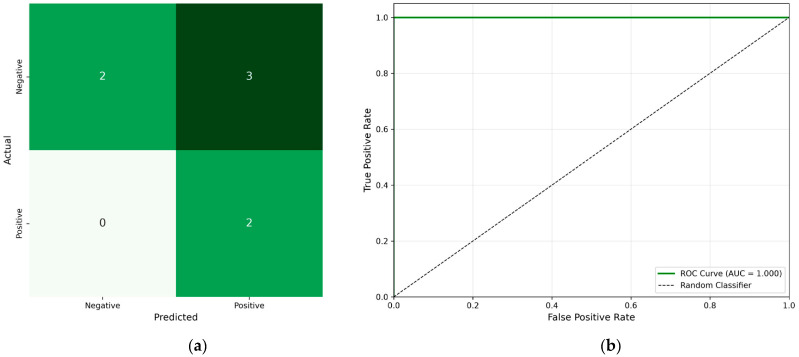
Performance of the final alternative fibroid-feature LR model for Cohort 2, as evaluated on the held-out test set. (**a**) Confusion matrix showing 2 true negatives, 2 true positives, 3 false positives, and 0 false negatives. (**b**) ROC curve demonstrating the model’s discriminative performance on the test set, with an AUC of 1.00.

In Cohort 2, the final selected model was the alternative fibroid-feature LR model trained with ROS. On the held-out test set, this model achieved an AUC of 1.00, F1-score for the preterm birth class of 0.57, recall of 1.00, precision of 0.40, accuracy of 0.57, Cohen’s kappa of 0.28, and Brier score of 0.16. The confusion matrix in Figure 5 shows two true negatives, two true positives, three false positives, and zero false negatives.

These test-set results should be interpreted cautiously because the held-out test subsets contained only three preterm birth events in Cohort 1 and two preterm birth events in Cohort 2.

#### 3.5.2. Bootstrap Confidence Intervals

**Figure 6:**
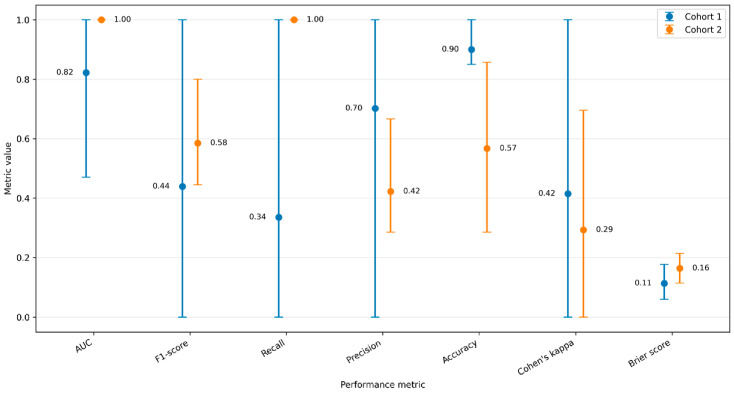
Bootstrap 95% confidence intervals for final held-out test-set performance metrics in Cohort 1 and Cohort 2. Points represent the mean bootstrap estimate for each performance metric, and vertical error bars represent the percentile-based 95% bootstrap confidence interval, calculated using the 2.5th and 97.5th percentiles of the bootstrap distribution. Metrics include AUC, F1-score, recall, precision, accuracy, Cohen’s kappa, and Brier score. For AUC, F1-score, recall, precision, accuracy, and Cohen’s kappa, higher values indicate better performance, whereas for the Brier score, lower values indicate better probabilistic accuracy.

Bootstrap confidence intervals for the final selected models are shown in Figure 6. The intervals showed substantial uncertainty for several test-set performance metrics, reflecting the small number of positive events in the held-out test sets.

In Cohort 1, the mean bootstrap AUC was 0.82, with a 95% confidence interval ranging from 0.47 to 1.00. Confidence intervals for F1-score, recall, precision, and Cohen’s kappa were wide, indicating substantial uncertainty around the test-set classification metrics. Accuracy was more stable, with a mean of 0.90 and a 95% confidence interval of 0.85 to 1.00, reflecting the predominance of term births in the test set. The mean bootstrap Brier score was 0.11, with a 95% confidence interval of 0.06 to 0.18.

In Cohort 2, the mean bootstrap AUC was 1.00, with a 95% confidence interval of 1.00 to 1.00. Recall also remained 1.00 across bootstrap samples. However, these estimates should not be interpreted as evidence of stable clinical performance because they were based on only two positive test-set cases. The mean F1-score was 0.59, with a 95% confidence interval of 0.44 to 0.80. Precision, accuracy, and Cohen’s kappa showed broader uncertainty, with mean values of 0.42, 0.57, and 0.29, respectively. The mean bootstrap Brier score was 0.16, with a 95% confidence interval of 0.11 to 0.21.

Overall, the bootstrap results support cautious interpretation of the final test-set performance in both cohorts, particularly given the very small number of preterm birth events in the held-out test subsets.

#### 3.5.3. Calibration of the Final Selected Models

Calibration results are presented in Supplementary Figures S1 and S2 and Supplementary Table S9. Supplementary Figure S1 shows the calibration plot for the final selected Cohort 1 model, and Supplementary Figure S2 shows the calibration plot for the final selected Cohort 2 model. Supplementary Table S9 reports the Brier score, calibration slope, calibration intercept, and the number of calibration bins.

In Cohort 1, the final selected model had a Brier score of 0.113, calibration slope of 0.699, and calibration intercept of −0.707. The calibration curve showed some increase in observed event fraction across predicted-probability bins, suggesting partial risk ordering. However, the curve remained below the ideal calibration line in the higher predicted-risk range, indicating overestimation of predicted risk.

In Cohort 2, the final selected model had a Brier score of 0.164, calibration slope of 104.444, and calibration intercept of −10.056. The calibration curve showed an abrupt increase in the highest predicted-probability bin, while the lower bins contained no observed positive cases. The extreme calibration slope and intercept indicate numerical instability rather than reliable evidence of good calibration.

Overall, calibration assessment remained limited in both cohorts. Cohort 1 showed partial risk ordering but some overestimation of predicted risk, whereas Cohort 2 showed highly unstable calibration estimates. These findings should be interpreted cautiously because of the very small number of positive events in the held-out test sets.

### 3.6. Model Interpretation

**Figure 7:**
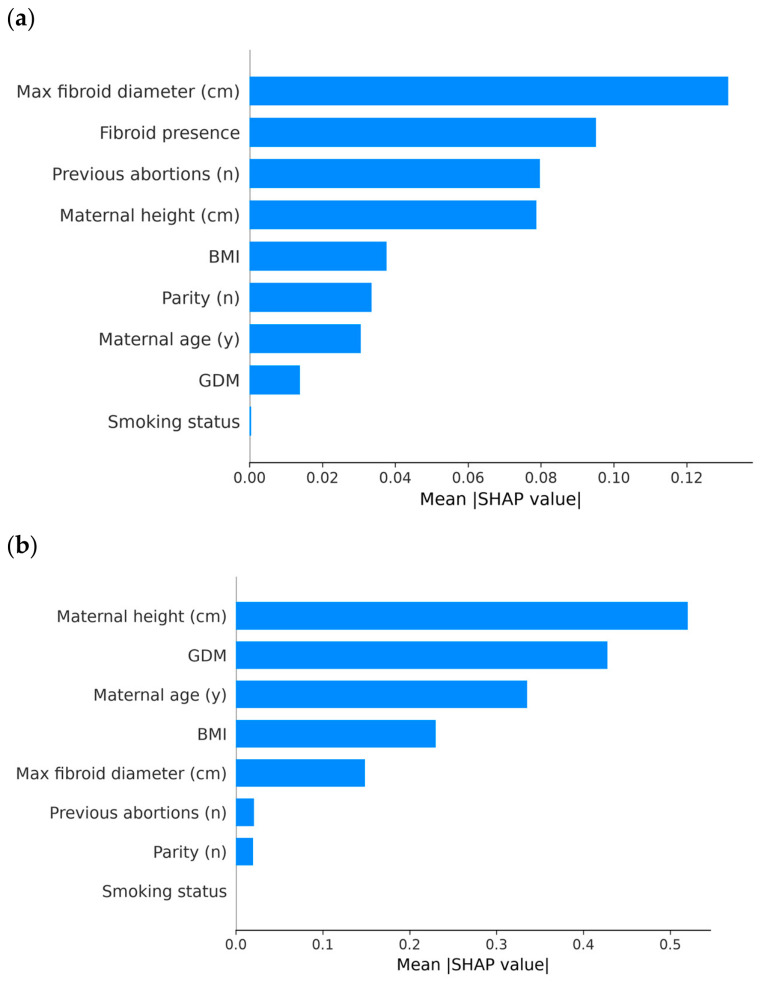
SHAP summary bar plots showing the global importance of predictors for preterm birth in Cohort 1 (**a**) and Cohort 2 (**b**). Feature importance is expressed as the mean absolute SHAP value across observations in the held-out validation set. In Cohort 1, maximum fibroid diameter was the most influential predictor, followed by fibroid presence, previous abortions, maternal height, BMI, parity, maternal age, GDM, and smoking status. In Cohort 2, maternal height was the most influential predictor, followed by GDM, maternal age, BMI, maximum fibroid diameter, previous abortions, parity, and smoking status. Abbreviations: BMI, body mass index; cm, centimeters; n, number; GDM, gestational diabetes mellitus; y, years.

Figure 7 presents the SHAP summary bar plots for the predictors of preterm birth in the two study cohorts, with (a) representing Cohort 1 and (b) representing Cohort 2. Cohort 1 included the full study population, comprising women both with and without fibroids, whereas Cohort 2 included only women with huge uterine fibroids.

In Cohort 1, maximum fibroid diameter was the most influential predictor, followed by fibroid presence, previous abortions, maternal height, BMI, parity, maternal age, GDM, and smoking status. In Cohort 2, maternal height showed the largest contribution, followed by GDM, maternal age, BMI, maximum fibroid diameter, previous abortions, parity, and smoking status. Thus, maximum fibroid diameter was the leading contributor in the full cohort, but not in the fibroid-only cohort, where maternal and pregnancy-related variables had larger SHAP contributions.

**Figure 8:**
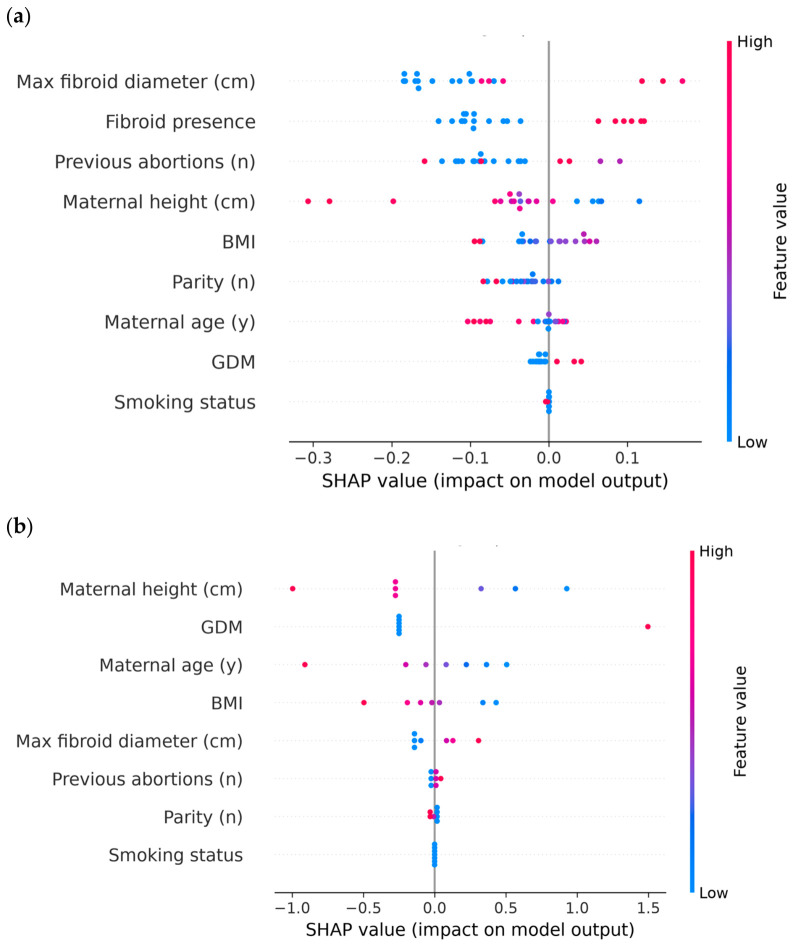
SHAP summary dot plots showing the distribution and direction of feature effects on the prediction of preterm birth in Cohort 1 (**a**) and Cohort 2 (**b**). Each dot represents one observation, positioned according to its SHAP value, with color indicating the relative feature value from low (blue) to high (pink). Positive SHAP values indicate a higher contribution to the predicted risk of preterm birth, whereas negative SHAP values indicate a lower contribution. In Cohort 1, maximum fibroid diameter and fibroid presence showed prominent contributions to model output. In Cohort 2, maternal height, GDM, maternal age, and BMI showed the largest contributions, while maximum fibroid diameter contributed to a lesser extent than in Cohort 1. Abbreviations: BMI, body mass index; cm, centimeters; n, number; GDM, gestational diabetes mellitus; y, years.

Figure 8 presents the SHAP summary dot plots for the prediction of preterm birth in the two study cohorts, with (a) representing Cohort 1 and (b) representing Cohort 2. In Cohort 1, maximum fibroid diameter and fibroid presence showed prominent contributions to model output. Higher values of maximum fibroid diameter and the presence of fibroids generally shifted predictions toward higher predicted risk, whereas lower values tended to shift predictions in the opposite direction. Previous abortions, maternal height, BMI, parity, maternal age, and GDM showed more heterogeneous effects across observations, while smoking status contributed minimally to model output.

In Cohort 2, maternal height showed the widest SHAP value distribution. Lower maternal height generally shifted predictions toward higher predicted risk, whereas higher maternal height tended to shift predictions in the opposite direction. GDM also contributed positively to predicted risk in the observed cases. Maternal age and BMI showed wide but heterogeneous SHAP distributions. Maximum fibroid diameter contributed to model output to a lesser extent than in Cohort 1, while previous abortions, parity, and smoking status showed relatively small effects.

Overall, the SHAP analyses indicate that fibroid-related variables contributed to model output, particularly in the full cohort. However, the relative importance of fibroid-related, maternal, obstetric, and pregnancy-related variables differed between cohorts. These SHAP findings should be interpreted as model-based feature contributions rather than causal effects, and should be viewed as supporting model interpretability and hypothesis generation rather than establishing causal mechanisms.

**Figure 9:**
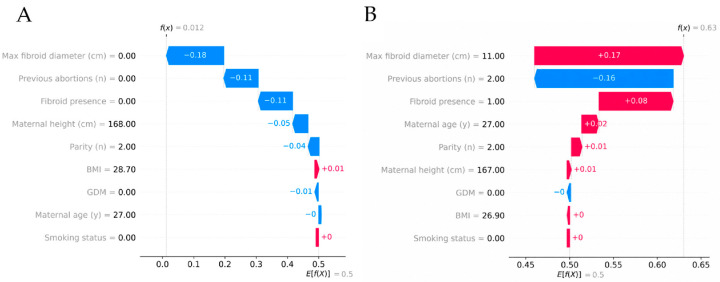
SHAP waterfall plots for two example profiles from the full cohort (*n* = 192). Profile (**A**) represents a term birth, and profile (**B**) represents a preterm birth. The plots show how individual maternal and fibroid-related features contributed to the predicted probability of preterm birth. Red bars indicate features that increased the predicted risk, whereas blue bars indicate features that decreased the predicted risk. Numbers within the bars represent feature-specific SHAP contributions. E[f(x)] represents the average predicted risk across the full cohort, and f(x) represents the predicted risk for the individual participant shown. Abbreviations: BMI, body mass index; cm, centimeters; n, number; GDM, gestational diabetes mellitus; y, years.

Figure 9 presents SHAP waterfall plots for two cases, illustrating individualized risk profiles for preterm birth. The examples demonstrate how the same set of predictors can contribute differently across subjects, with each plot showing the direction and magnitude of feature contributions for a single participant.

In profile A, which represents a term birth, the model assigned a low predicted probability of preterm birth (0.012). The main contributors toward lower predicted risk were the absence of fibroids, a maximum fibroid diameter of 0 cm, no previous abortions, a maternal height of 168 cm, and a parity of two. Smaller negative contributions were also observed for the absence of gestational diabetes mellitus (GDM) and a maternal age of 27 years, whereas a BMI of 28.7 kg/m^2^ made only a minimal positive contribution. Overall, these factors shifted the prediction toward a very low estimated risk of preterm birth (Figure 9A).

In profile B, which represents a preterm birth, the model assigned a higher predicted probability of preterm birth (0.63). The strongest positive contributor was a maximum fibroid diameter of 11 cm, followed by fibroid presence. Smaller positive contributions were observed for a maternal age of 27 years, a parity of two, and a maternal height of 167 cm. In contrast, two previous abortions contributed to a negative direction and partially offset the predicted risk, while the absence of GDM, a BMI of 26.9 kg/m^2^, and non-smoking status had minimal effects. Together, these contributions resulted in a substantially higher predicted risk of preterm birth (Figure 9B).

## 4. Discussion

### 4.1. Principal Findings

In this stratified cohort analysis, we evaluated ML models for preterm birth prediction using detailed fibroid phenotyping in both a full cohort and a fibroid-only cohort. The principal finding was that fibroid-related characteristics contributed predictive information beyond non-fibroid variables and conventional clinical benchmark models.

In the full cohort, the strongest validation performance was observed for the alternative fibroid-feature RF model. Compared with the best non-fibroid ML configuration (Figure 3), the F1-score increased from 0.40 to 0.67, corresponding to an absolute improvement of 0.27. Compared with the clinical benchmark configuration, the improvement was larger, with the F1-score increasing from 0.27 to 0.67. Accuracy also increased from 0.85 in the non-fibroid configuration and 0.45 in the clinical benchmark to 0.90 in the alternative fibroid-feature ML configuration. These validation-stage improvements suggest that structured fibroid-related information improved classification performance in the full cohort.

In the fibroid-only cohort, the strongest validation performance was observed for the alternative fibroid-feature LR model. Compared with the full fibroid-feature ML configuration (Figure 3), the F1-score increased from 0.67 to 0.80, corresponding to an absolute improvement of 0.13, and accuracy increased from 0.71 to 0.86. Compared with the clinical benchmark configuration, the F1-score increased from 0.50 to 0.80, and accuracy increased from 0.43 to 0.86. The alternative fibroid-feature configuration achieved the same F1-score and accuracy as the best non-fibroid ML configuration, but had a higher AUC of 0.90 versus 0.80 and a lower Brier score of 0.14 versus 0.19. These results suggest that detailed fibroid descriptors may contribute additional predictive information even among women who all share the underlying exposure of a huge uterine fibroid.

The final held-out test-set evaluation (Table 2) showed preliminary discriminatory signal but also emphasized the instability expected in a small dataset. In the full cohort, the final alternative fibroid-feature RF model achieved an F1-score of 0.50, accuracy of 0.90, recall of 0.33, AUC of 0.82, Cohen’s kappa of 0.46, and Brier score of 0.11. In the fibroid-only cohort, the final alternative fibroid-feature LR model achieved an F1-score of 0.57, accuracy of 0.57, recall of 1.00, AUC of 1.00, Cohen’s kappa of 0.28, and Brier score of 0.16. However, because the held-out test sets contained only three preterm birth events in the full cohort and two in the fibroid-only cohort, these estimates should be interpreted cautiously. In particular, the high AUC and recall in the fibroid-only cohort should not be interpreted as evidence of stable clinical performance.

The interpretability analyses further supported the relevance of fibroid phenotyping, although the relative importance of predictors differed between cohorts. In the SHAP summary bar plots, maximum fibroid diameter was the most influential predictor in the full cohort, followed by fibroid presence, previous abortions, maternal height, BMI, parity, maternal age, GDM, and smoking status. In the fibroid-only cohort, maternal height showed the largest contribution, followed by GDM, maternal age, BMI, maximum fibroid diameter, previous abortions, parity, and smoking status (Figure 7). Thus, maximum fibroid diameter was the leading contributor in the full cohort but not in the fibroid-only cohort, where maternal and pregnancy-related variables showed larger SHAP contributions.

The SHAP dot plots showed that fibroid-related variables contributed to model output, particularly in the full cohort. In Cohort 1, maximum fibroid diameter and fibroid presence showed prominent contributions to predicted risk, while other predictors showed smaller or more heterogeneous effects across observations. In Cohort 2, maternal height, GDM, maternal age, and BMI showed the largest SHAP distributions, whereas maximum fibroid diameter contributed to a lesser extent than in the full cohort (Figure 8). These SHAP findings should be interpreted as model-based feature contributions rather than causal effects. Although the contribution of fibroid-related variables is clinically plausible, the contribution of individual predictors may reflect correlations among predictors, residual confounding, sample composition, or center-specific patterns of care. Therefore, the SHAP analysis should be viewed as supporting model interpretability and hypothesis generation, rather than establishing causal mechanisms.

### 4.2. Comparison with Prior Studies

Several ML studies have predicted preterm birth using routine electronic health record or administrative data without incorporating fibroids or other direct uterine structural markers. Abraham et al. developed gradient-boosted decision tree models in a large EHR cohort of 35,282 deliveries and reported a ROC-AUC of approximately 0.75 at 28 weeks of gestation, with external validation in an independent cohort of 5978 deliveries; the comparison model based on known clinical risk factors included variables such as race, age, diabetes, hypertensive disorders, and cervical abnormalities, but fibroids were not listed among the predictors [9]. Similarly, Khan et al. analyzed 3509 pregnancies and found that XGBoost achieved the best performance, with AUCs of 0.735 in parous women and 0.723 in nulliparous women; their feature set included multiple maternal and clinical risk factors, but fibroids or myomas were not reported among them [10]. Likewise, Yu et al. developed prediction models in 22,603 singleton pregnancies and reported that CatBoost performed best after 26 weeks of gestation, with an AUC of 0.70, using antenatal surveillance features rather than fibroid-related variables [30].

In contrast, a smaller group of studies explicitly included fibroids, myomas, or related uterine structural conditions in their models. Qian et al. analyzed 36,378 pregnancies, with external validation in 10,367 women, and included uterine fibroids among candidate risk factors; fibroids were more common in the preterm group and were retained among the statistically significant predictors used for model construction. In that study, the best conventional ML model was RF with an AUC of 0.826, whereas a dynamic Long Short-Term Memory (LSTM) model based on serial cervical length data achieved the best overall performance, with an AUC of 0.851 [11]. Similarly, Zhang et al. included “pregnancy complicated with uterine fibroids” among candidate variables in a cohort of 5411 pregnancies, but this variable was not statistically significant and was not retained in the final model, despite the AdaBoost model achieving an AUC of approximately 0.93 [13]. In Lee and Ahn’s study, the model included “myomas and adenomyosis” in 596 obstetric patients, but their contribution was minimal, with very low variable importance compared with maternal comorbidities and obstetric history [2].

An important distinction between our study and most previous ML studies is that we did not model fibroids only as a simple present/absent condition. Instead, we evaluated structured fibroid phenotyping across several feature-set configurations and separately analyzed a fibroid-only cohort in order to assess prediction within the population in which fibroid characteristics are most clinically relevant. This approach provides a more fibroid-centered framework and may explain why fibroid-related variables were retained in the selected fibroid-feature configurations and contributed to model output, although their relative importance differed between cohorts.

### 4.3. Clinical Interpretation and Implications

The clinical implication of these findings is that fibroid phenotype should not be treated only as a binary “present/absent” variable when assessing obstetric risk. In the full cohort, structured fibroid information improved validation-stage performance, suggesting that details such as fibroid size and selected anatomical descriptors may help identify women who could benefit from closer antenatal surveillance. This is clinically relevant because preterm birth is a common and important obstetric outcome, and even modest improvements in early risk stratification may influence monitoring intensity, referral patterns, and obstetric planning [4]. The approach presented in the current study addresses a gap in current preterm birth prediction research, where most models either omit fibroids entirely or encode them inadequately [9,10,11].

Early identification of women at increased risk for preterm birth may support closer antenatal surveillance and more individualized counseling regarding potential complications and delivery planning. For pregnant women with huge uterine fibroids, these findings may contribute to more individualized risk assessment [1,3,4].

In the fibroid-only cohort, the alternative fibroid-feature configuration showed the strongest overall validation profile, although its advantage over the non-fibroid ML configuration was mainly reflected in the AUC and Brier score rather than the F1-score or accuracy. SHAP analyses also showed that maximum fibroid diameter remained among the contributing predictors, but maternal and pregnancy-related variables had larger contributions in this cohort. These findings suggest that detailed fibroid descriptors may add predictive information, but their contribution may vary depending on the analytical cohort and the available clinical context.

Overall, these results align with meta-analytic evidence that fibroids are associated with preterm birth [3,4] and support further investigation of structured fibroid phenotyping in future preterm birth prediction models. Larger prospective studies with standardized fibroid characterization and external validation are required before clinical implementation.

### 4.4. Limitations

This study has several limitations. First, its retrospective single-center design and relatively small sample size, particularly in the fibroid-only cohort, may limit statistical power and the stability of model estimates. The small validation and test subsets further increase the uncertainty of performance estimates, as metrics such as precision, recall, F1-score, and Cohen’s kappa may be sensitive to the classification of only a small number of cases. In addition, although only predictors available before the outcome were included, the retrospective nature of the dataset may limit precise control over the timing and completeness of variable collection.

Second, although strict train/validation/test separation and leakage-free preprocessing procedures were applied, the models were developed and evaluated without external validation, limiting their generalizability to other populations and clinical settings. Third, the use of detailed fibroid-related variables increases the interpretability of the analysis but may also introduce redundancy among related fibroid encodings, such as fibroid size, size categories, type, and location. Although redundancy assessment and feature-set simplification were performed, residual correlations among predictors may still affect model estimation and interpretation.

Finally, SHAP-based interpretation is limited by the small sample size and retrospective design, and SHAP values should not be interpreted as causal effects. Therefore, these findings should be considered preliminary and require validation in larger cohorts with standardized data collection and external validation before clinical implementation.

### 4.5. Future Research Directions

Future research should validate these findings in larger and more diverse populations, including multicenter cohorts with standardized ultrasound-based fibroid characterization. Larger sample sizes and higher numbers of preterm birth events will be needed to improve model stability, evaluate calibration more reliably, and determine whether detailed fibroid phenotype improves prediction beyond conventional clinical variables.

Future studies should also examine whether fibroid phenotype improves prediction at clinically meaningful gestational time points and whether model predictions can be recalibrated across centers. Ultimately, the role of detailed fibroid phenotyping should be tested as part of transparent, externally validated prediction models before being considered for clinical decision-support use.

## 5. Conclusions

This study suggests that detailed phenotyping of huge uterine fibroids may provide predictive information for preterm birth beyond conventional clinical variables and a simple binary fibroid/no-fibroid classification. In validation analyses, the alternative fibroid-feature configuration was selected as the best-performing configuration in both cohorts according to the predefined selection criterion. In the full cohort, fibroid-feature ML improved performance compared with both the non-fibroid ML configuration and the conventional clinical benchmark. In the fibroid-only cohort, the alternative fibroid-feature configuration showed the strongest overall validation profile.

Model interpretation suggested that fibroid-related variables contributed to model output, but their relative importance differed between cohorts. Maximum fibroid diameter was the leading contributor in the full cohort, whereas in the fibroid-only cohort, maternal and pregnancy-related variables showed larger SHAP contributions, with maximum fibroid diameter remaining among the contributing predictors.

However, the findings should be interpreted as exploratory and hypothesis-generating. The study was retrospective and single-center, with a small sample size, limited number of outcome events, low events-per-variable estimates, wide or unstable bootstrap confidence intervals, limited calibration, and no external validation. Therefore, the models should not be considered clinically deployable prediction tools at this stage.

Overall, the results support further investigation of detailed fibroid phenotype, particularly fibroid size and selected anatomical descriptors, as a candidate feature domain in future preterm birth prediction studies. Larger multicenter cohorts with standardized fibroid characterization, adequate event counts, calibration assessment, and external validation are required to determine whether detailed fibroid phenotyping can improve clinically useful preterm birth risk stratification.

## Figures and Tables

**Table 1:** Candidate baseline predictors considered before redundancy-based feature reduction, grouped by category.

Category	Features
Fibroid-related characteristics (*n* = 9)	Study group Fibroid presence Max fibroid diameter (cm) Fibroid size (tertile) ^T^ Fibroid size (cutoff) ^U^ Fibroid size (median) Fibroid type Fibroid anatomical location Fibroid location category
Maternal anthropometrics (*n* = 5)	Maternal height (cm) Maternal weight (kg) BMI Obesity status BMI category
Obstetrics (*n* = 5)	Gravidity Parity (*n*) Parity category High gravidity Previous abortions (*n*)
Maternal demographics (*n* = 3)	Maternal age (y) Maternal age group Education level
Current pregnancy uteroplacental insufficiency risk factors (*n* = 2)	Smoking status GDM

^T^ The distribution of continuous values of all fibroids was split into three tertiles (10.0–10.1 cm, 10.1–12.1 cm, and >12.1 cm). ^U^ The cutoff was based on maximum fibroid diameter at the upper tertile (>12.1 cm).

**Table 2:** Test-set performance of the two final models for preterm birth prediction.

Cohort	Final Model	F1-Score	Accuracy	Precision	Recall	AUC	Cohen’s Kappa	Brier Score
Cohort 1	RF	0.50	0.90	1.00	0.33	0.82	0.46	0.11
Cohort 2	LR	0.57	0.57	0.40	1.00	1.00	0.28	0.16

## Data Availability

The data presented in this study are available from the corresponding author upon reasonable request. Due to privacy and ethical restrictions, the data are not publicly available. An anonymized version of the dataset can be shared with qualified researchers for research purposes.

## Supplementary Materials

The following supporting information can be downloaded at: https://www.mdpi.com/article/10.3390/diagnostics16142242/s1, Table S1: Python package versions used in this study; Table S2: Cohort sizes, preterm birth events, candidate predictors, retained predictors, selected features, and events-per-variable estimates by cohort and feature-set configuration; Table S3: Pairwise redundancy assessment among candidate predictors using Spearman correlation, Cramér’s V, and phi coefficients; Table S4: Candidate predictors retained after redundancy assessment and before feature selection by analysis configuration; Table S5: Final selected features after full-training-set refit for each cohort, configuration, model, and resampling strategy; Table S6: Feature-selection results and fold-level performance metrics across cross-validation folds; Table S7: Best hyperparameters selected for each ML pipeline across cohorts, configurations, models, and resampling strategies; Table S8: Held-out validation performance of all candidate pipelines across cohorts and feature-set configurations; Table S9: Brier score, calibration slope, calibration intercept, and number of calibration bins for the final selected models in each cohort; Figure S1: Calibration plots for the final selected models in Cohort 1; Figure S2: Calibration plots for the final selected models in Cohort 2.

## Abbreviations

The following abbreviations are used in this manuscript:

AUC	Area under the receiver operating characteristic curve
BMI	Body mass index
CV	Cross-validation
GDM	Gestational diabetes mellitus
EPV	Calculated events-per-variable
LightGBM	Light Gradient Boosting Machine
LR	Logistic Regression
ML	Machine learning
RF	Random Forest
ROC	Receiver operating characteristic
ROS	Random oversampling
RFE	Recursive feature elimination
SHAP	SHapley Additive exPlanations
SMOTENC	Synthetic Minority Oversampling Technique for Nominal and Continuous Features

## Appendix A

**Table A1:** Descriptive characteristics of the study cohorts, including sample size, outcome counts, maternal baseline variables, and fibroid-related characteristics. Continuous variables are presented as mean and standard deviation. Fibroid-specific characteristics are reported for the relevant fibroid-only cohort.

	Cohort 1	Cohort 2
*N*	192	64
Number of preterm cases	25	19
Maternal age (y)		
Mean	31.88	34.25
SD	5.54	5.28
Maternal height (cm)		
Mean	163.91	166.66
SD	5.89	4.79
Maternal weight (kg)		
Mean	77.03	80.81
SD	12.13	11.37
Maternal BMI		
Mean	28.99	29.90
SD	4.20	3.91
Previous abortions		
Mean	0.64	0.92
SD	0.86	0.86
Gravidity		
Mean	3.06	3.35
SD	1.64	1.35
Parity		
Mean	2.91	2.28
SD	1.83	0.89
Max fibroid diameter		
Mean		11.79
SD		2.09
Fibroid type, *n* (%)		
Intramural		32 (50.0%)
Subserosal		23 (35.9%)
Previa		7 (10.9%)
Cervical		2 (3.1%)
Anatomical location, *n* (%)		
Fundus		41 (64.1%)
Previa/Cervical		9 (14.1%)
Lower uterine segment		7 (10.9%)
Posterior wall		4 (6.3%)
Anterior wall		3 (4.7%)

## References

[1] World Health Organization 152 Million Babies Born Preterm in the Last Decade. https://www.who.int/news/item/09-05-2023-152-million-babies-born-preterm-in-the-last-decade.

[2] Lee K.S., Ahn K.H. (2019). Artificial neural network analysis of spontaneous preterm labor and birth and its major determinants. J. Korean Med. Sci..

[3] Landman A.J.E.M.C., Don E.E., Vissers G., Ket H.C.J., Oudijk M.A., de Groot C.J.M., de Boer M.A. (2022). The risk of preterm birth in women with uterine fibroids: A systematic review and meta-analysis. PLoS ONE.

[4] Li H., Hu Z., Fan Y., Hao Y. (2024). The influence of uterine fibroids on adverse outcomes in pregnant women: A meta-analysis. BMC Pregnancy Childbirth.

[5] Ciavattini A., Di Giuseppe J., Stortoni P., Montik N., Giannubilo S.R., Litta P., Islam M.S., Tranquilli A.L., Reis F.M., Ciarmela P. (2013). Uterine Fibroids: Pathogenesis and Interactions with Endometrium and Endomyometrial Junction. Obstet. Gynecol. Int..

[6] Navarro A., Bariani M.V., Yang Q., Al-Hendy A. (2021). Understanding the Impact of Uterine Fibroids on Human Endometrium Function. Front. Cell Dev. Biol..

[7] Lam S.-J., Best S., Kumar S. (2014). The impact of fibroid characteristics on pregnancy outcome. Am. J. Obstet. Gynecol..

[8] Dayanan R., Duygulu Bulan D., Ayas Ozkan M., Karabay G., Seyhanli Z., Beydilli Sural E., Basmaz F., Kunt S., Celen S. (2025). Influence of uterine fibroid size on perinatal and neonatal outcomes: A single-centre cohort of 651 pregnancies. BMC Pediatr..

[9] Abraham A., Le B., Kosti I., Straub P., Velez-Edwards D.R., Davis L.K., Capra J.A. (2022). Dense phenotyping from electronic health records enables machine learning-based prediction of preterm birth. BMC Med..

[10] Khan W., Zaki N., Ghenimi N., Ahmad A., Bian J., Masud M.M., Ahmed L.A. (2023). Predicting preterm birth using explainable machine learning in a prospective cohort of nulliparous and multiparous pregnant women. PLoS ONE.

[11] Qian L., Jia H., Chang Z., Hu Y., Chen C., Li X., Zhang H. (2025). Predicting the risk of preterm birth with machine learning and electronic health records in China. BMC Med. Inform. Decis. Mak..

[12] Wong K., Tessema G.A., Chai K., Pereira G. (2022). Development of prognostic model for preterm birth using machine learning in a population-based cohort of Western Australia births between 1980 and 2015. Sci. Rep..

[13] Zhang Y., Du S., Hu T., Xu S., Lu H., Xu C., Zhu X. (2023). Establishment of a model for predicting preterm birth based on the machine learning algorithm. BMC Pregnancy Childbirth.

[14] Lundberg S.M., Lee S.I. A unified approach to interpreting model predictions. Proceedings of the 31st International Conference on Neural Information Processing Systems.

[15] Collins G.S., Moons K.G.M., Dhiman P., Riley R.D., Beam A.L., Van Calster B., Ghassemi M., Liu X., Reitsma J.B., van Smeden M. (2024). TRIPOD+AI statement: Updated guidance for reporting clinical prediction models that use regression or machine learning methods. BMJ.

[16] World Health Organization (2000). Obesity: Preventing and Managing the Global Epidemic.

[17] Chambers G.M., Venetis C.A., Jorm L.R., Stavrou E.P., Vajdic C.M. (2020). Parity: A key measure of confounding in data-linkage studies of outcomes after medically assisted reproduction. Int. J. Popul. Data Sci..

[18] Dormann C.F., Elith J., Bacher S., Buchmann C., Carl G., Carré G., García Márquez J.R., Gruber B., Lafourcade B., Leitão P.J. (2013). Collinearity: A review of methods to deal with it and a simulation study evaluating their performance. Ecography.

[19] Nick T.G., Campbell K.M., Ambrosius W.T. (2007). Logistic regression. Topics in Biostatistics.

[20] Firth D. (1993). Bias reduction of maximum likelihood estimates. Biometrika.

[21] Hoffman M.K. (2021). Prediction and prevention of spontaneous preterm birth: ACOG Practice Bulletin, Number 234. Obstet. Gynecol..

[22] Tibshirani R. (1996). Regression shrinkage and selection via the lasso. J. R. Stat. Soc. Ser. B Stat. Methodol..

[23] Breiman L. (2001). Random forests. Mach. Learn..

[24] Ke G., Meng Q., Finley T., Wang T., Chen W., Ma W., Ye Q., Liu T.-Y. (2017). LightGBM: A Highly Efficient Gradient Boosting Decision Tree. Advances in Neural Information Processing Systems.

[25] Peng H., Long F., Ding C.H.Q. (2005). Feature Selection Based on Mutual Information: Criteria of Max-Dependency, Max-Relevance, and Min-Redundancy. IEEE Trans. Pattern Anal. Mach. Intell..

[26] Chen X.W., Jeong J.C. Enhanced recursive feature elimination. Proceedings of the Sixth International Conference on Machine Learning and Applications (ICMLA 2007).

[27] Batista G.E., Prati R.C., Monard M.C. (2004). A study of the behavior of several methods for balancing machine learning training data. ACM SIGKDD Explor. Newsl..

[28] Efron B., Tibshirani R. (1986). Bootstrap methods for standard errors, confidence intervals, and other measures of statistical accuracy. Stat. Sci..

[29] Van Calster B., McLernon D.J., van Smeden M., Wynants L., Steyerberg E.W., On behalf of Topic Group ‘Evaluating Diagnostic Tests and Prediction Models’ of the STRATOS Initiative (2019). Calibration: The Achilles heel of predictive analytics. BMC Med..

[30] Yu Q.Y., Lin Y., Zhou Y.R., Yang X.J., Hemelaar J. (2024). Predicting risk of preterm birth in singleton pregnancies using machine learning algorithms. Front. Big Data.

