# Supplementary figures and images for: Machine-Learning-Based Prediction of Preterm Birth in Women with Huge Uterine Fibroids: A Stratified Cohort Analysis
Source: Diagnostics (Basel). 2026 Jul 17;16(14):2242. doi: 10.3390/diagnostics16142242 (PMC13409408; doi:10.3390/diagnostics16142242)

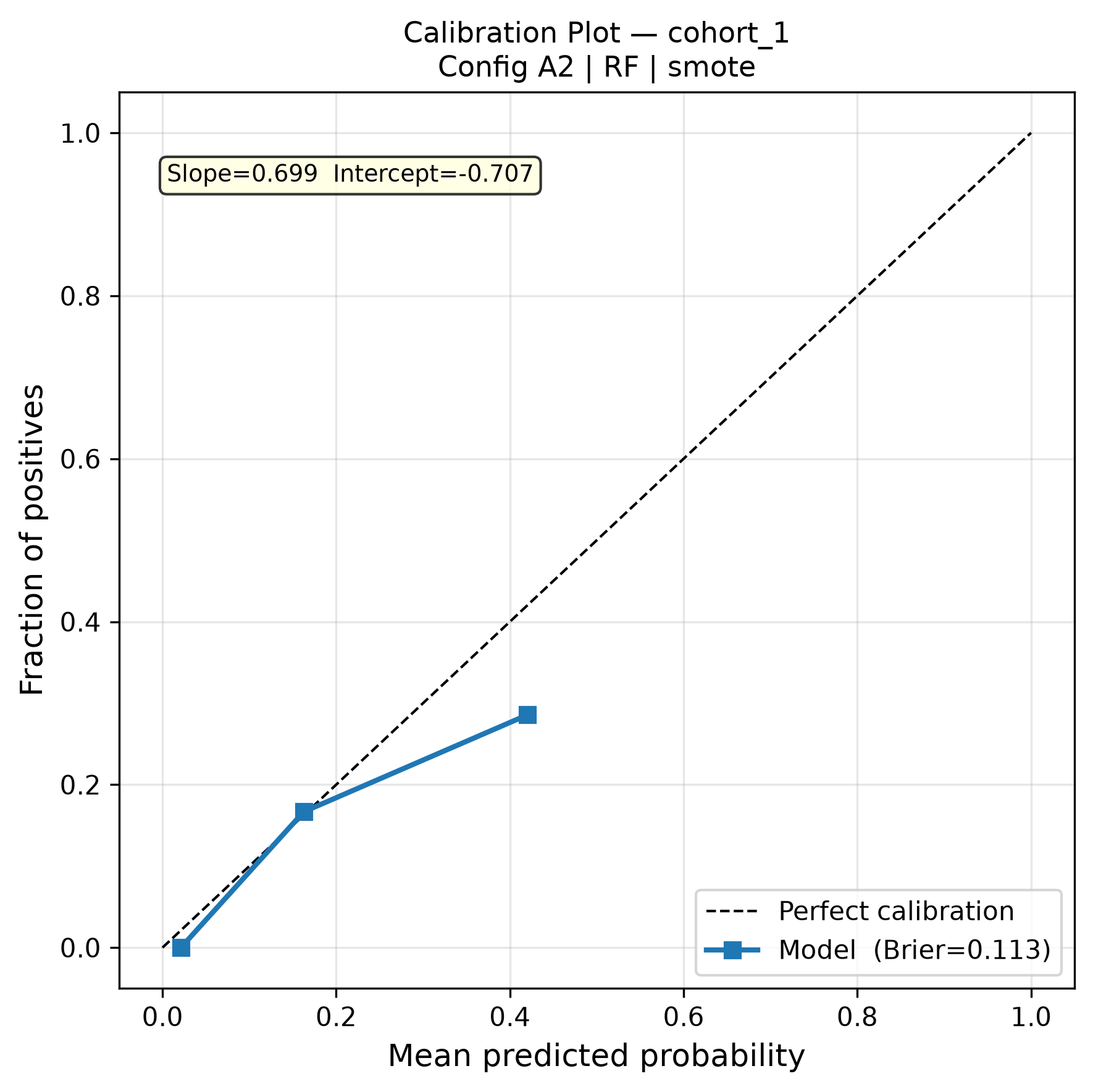

Supplement: Supplementary file 1 [file diagnostics-16-02242-s001.zip › Supplementary Figure S1.png]

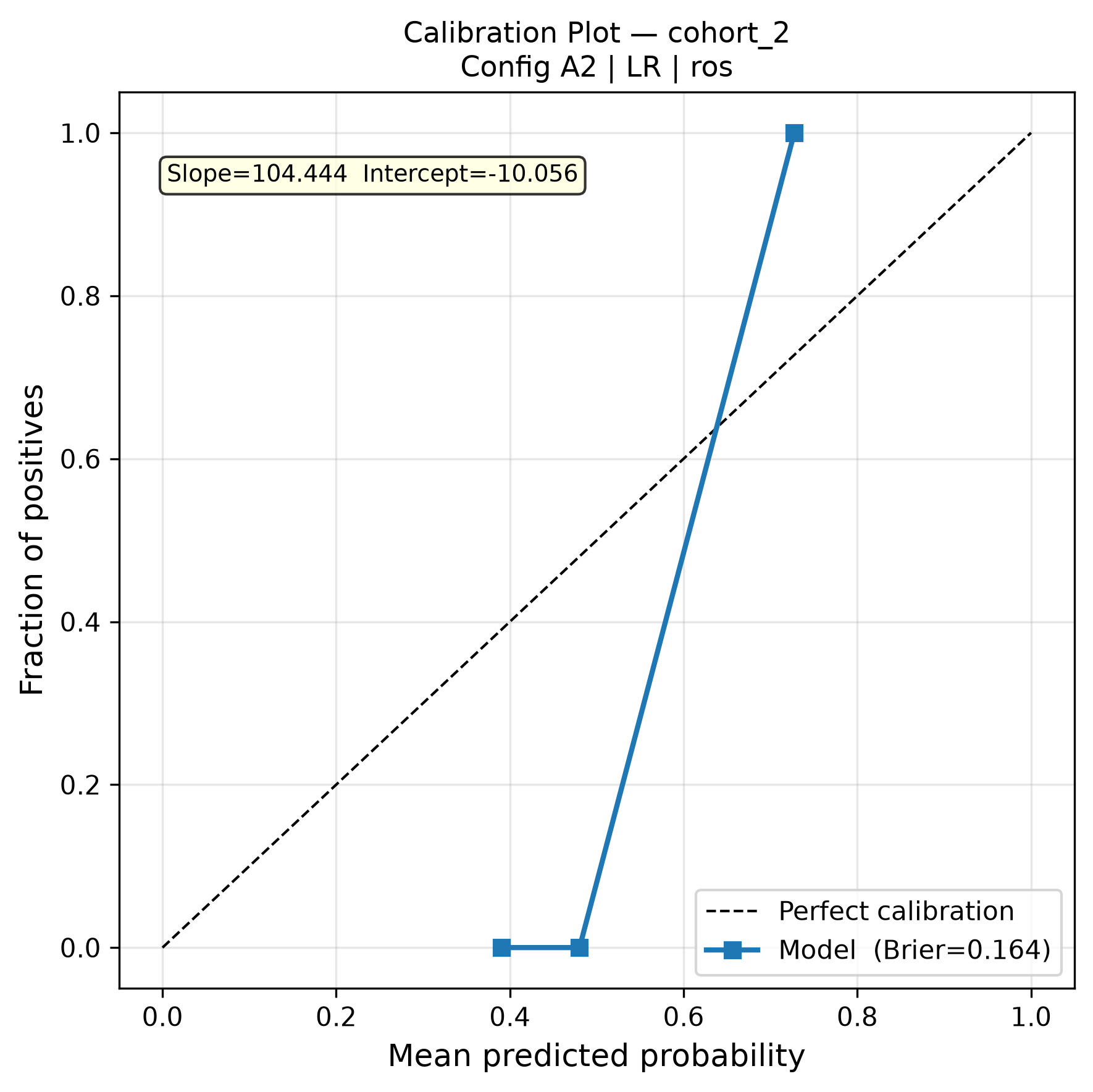

Supplement: Supplementary file 1 [file diagnostics-16-02242-s001.zip › Supplementary Figure S2.png]
